# Modulating Immune Response in Viral Infection for Quantitative Forecasts of Drug Efficacy

**DOI:** 10.3390/pharmaceutics15010167

**Published:** 2023-01-03

**Authors:** Bárbara Costa, Nuno Vale

**Affiliations:** 1 OncoPharma Research Group, Center for Health Technology and Services Research (CINTESIS), Rua Doutor Plácido da Costa, 4200-450 Porto, Portugal; 2 CINTESIS@RISE, Faculty of Medicine, University of Porto, Alameda Professor Hernâni Monteiro, 4200-319 Porto, Portugal; 3 Department of Community Medicine, Information and Health Decision Sciences (MEDCIDS), Faculty of Medicine, University of Porto, Rua Doutor Plácido da Costa, 4200-450 Porto, Portugal

**Keywords:** mechanistic models, viral infection, immune system, antiretroviral drug, drug efficacy

## Abstract

The antiretroviral drug, the total level of viral production, and the effectiveness of immune responses are the main topics of this review because they are all dynamically interrelated. Immunological and viral processes interact in extremely complex and non-linear ways. For reliable analysis and quantitative forecasts that may be used to follow the immune system and create a disease profile for each patient, mathematical models are helpful in characterizing these non-linear interactions. To increase our ability to treat patients and identify individual differences in disease development, immune response profiling might be useful. Identifying which patients are moving from mild to severe disease would be more beneficial using immune system parameters. Prioritize treatments based on their inability to control the immune response and prevent T cell exhaustion. To increase treatment efficacy and spur additional research in this field, this review intends to provide examples of the effects of modelling immune response in viral infections, as well as the impact of pharmaceuticals on immune response.

## 1. Introduction

Humans can contract a variety of viruses that have serious negative effects on their health and economy. Acute infections are caused by viruses, such as the rhinovirus, and influenza A or B viruses [1], whereas chronic infections are caused by others, such as the human immunodeficiency virus (HIV), Epstein-Barr virus (EBV), and hepatitis C virus (HCV) [2]. Various disease etiologists and levels of severity of viral infections exist, ranging from asymptomatic to fatal. Additionally, several viruses, including those that cause cancer, autoimmune diseases, and Alzheimer’s disease, such as EBV, human papillomavirus (HPV), or herpes simplex virus (HSV), may put a host at risk for coinfection with other pathogens [3].

In comparison with other pathogens, it is difficult to control viral-associated disorders because there is no one, all-encompassing method to control viruses due to the tremendous diversity in viruses’ epidemiology and pathogenicity. Even when prophylactic or therapeutic alternatives are available, inducing protective immunity may not always be successful and there may be decreased, time-dependent efficacy in single- or multi-pathogen infections. A lack of understanding of how host defense mechanisms restrict viral spread, how different viral components counteract these defense mechanisms, and how these relate to illness outcomes has impeded the development of new preventative and therapeutic measures. Numerous quantitative data have been generated recently because of improvements in multiparameter flow cytometry, high-throughput technologies and the SARS-CoV-2 pandemic, which pushed forward a lot of knowledge. These advancements have also brought attention to the need for new theoretical models that can explain complex biological interactions. Mathematical models have been developed to assess the several aspects of viral infection, since infection kinetics, to virus replication, mechanisms of viral persistence and control by host immune responses, as well as evaluate the clinical potential of various antiviral therapies [4,5]. These models have been curated and applied to in silico experiments to develop the creation of new hypotheses. Additionally, clinical dose-efficacy response and pharmacokinetic/pharmacodynamic (PK/PD) studies can be assisted using modelling and simulation platforms, which provide simulations for both viral dynamics and treatment efficacy [6]. This review aims to exemplify the impact of modulating the immune response in viral infections to improve drug efficacy.

## 2. Therapeutic Options for Viral Infections

For most viral infections there is no treatment to fight the virus, the patient can only hope for their immune system to fight back, and treatments can only take care of the symptoms. However, there are some antiviral medicines for some viruses and vaccines that help prevent harmful diseases [7]. Even though each virus has a unique biology that underlies infection, they all share several essential stages that can be used as targets for drug development. For instance, viral entrance is the first stage of viral infection. Specific receptors or attachment factors bind to and interact with proteins on the viral exterior, which may be proteins, glycans, or lipids (capsid or envelope) [8]. Through endocytic pathways, followed by trafficking through endosomes and lysosomes or by fusing at the plasma membrane, virus particles are frequently absorbed by the body as a result of these interactions. To infect cells productively, viruses need to escape from their vesicles (either endosomal escape or viral fusion can accomplish this). The genome is copied, translated (typically into intricate polyproteins that are processed by viral or host proteases), and, if necessary, transcribed into mRNA. Although some viruses also make use of host polymerases, many recently found RNA viruses have their own polymerases encoded. Although viral translation is controlled differently from cell translation, viruses always use host translation machinery. After the creation of structural proteins and genomes, viral particles are put together, which is then followed, for example, by cell lysis, at which time the cycle restarts. These stages in the overall viral life cycle can all be targeted by different drugs [9].

Table 1 presents different types of antiviral drugs used to fight viruses. Direct-acting antivirals, such as receptor decoys, nucleoside analogues, viral protease inhibitors, viral translation inhibitors, and antibodies, focus on the virus itself. The parts of the host cell necessary for a successful viral infection are targeted by host-factor antivirals, such as: antibodies that bind the receptor, endocytosis inhibitors, host protease inhibitors, lipidomic reprogramming medicines, and kinase inhibitors. Both direct-acting and host-factor treatments can be widely used if the antiviral targets conserved genes/motifs within a virus family or if several virus family members co-opt the same host pathways to promote virus replication and/or pathogenesis [10,11]. However, due to the biological pathways in both infected and healthy cells being disrupted, host-factor therapies may be more hazardous.

Viral infection treatments can work in a variety of ways. For instance, monoclonal antibodies and interferons can enhance and upregulate immune responses, respectively. Drugs can minimize the severity of symptoms caused by infection or a hyper-immune response as well as stimulate cellular responses for the more efficient clearance of virus-infected cells [12]. Antibodies and small chemicals can also directly interfere with the viral life cycle (e.g., by locating host kinases to target, kinase inhibitors alter the cellular environment). Multiple treatment modalities at various stages of infection may be needed to create medicines that reduce the length and severity of sickness. The selection of medications, as well as their dosing and timing, may need to be individualized due to patient-to-patient variability. Therefore, a way to select the best therapeutic option is understanding the innate immune response to viral infection, followed by disease severity of disease.

A better framework for drug development, repurposing, clinical trial design, and therapeutic optimization and personalization is required, as the SARS-CoV-2 pandemic has shown. Mechanics-based computational models may be crucial in creating these frameworks. The mechanisms of cellular immune responses to the virus, viral distribution and replication, and patient-to-patient heterogeneity in responses will be further discussed. Mechanistic models, which consider viral entry, replication in target cells, viral spread in the body, immune response, and the complex factors involved in tissue and organ damage and recovery, can also shed light on these mechanisms.

## 3. Mechanistic Models

Mechanistic models are useful to untangle the complex system of virus, cytokines, and immune cells by considering several factors, such as viral entry, replication in target cells, viral spread in the body, immune response, and other complex factors involved in tissue/organ damage and recovery. Temporal interactions coexist with time-dependent risk factors and patients multimorbidity, which must also be accounted for as infection progresses. Since all this needs to be modelled, it is necessary to separate each model and then integrate everything into a multiscale model [13].

Clinical studies of viral load performed on individual patients’ blood samples, immune cell or cytokine profile all provide snapshots of the viral infection [14], and we can recognize typical patterns of infection progression when we combine these data. The depth and frequency of these measures, however, are frequently insufficient to accurately predict everyone’s immune response and thus maximize the effectiveness of individualized treatments. We still have a limited knowledge of the mechanisms underlying why some people react to a virus with minor symptoms while others experience severe sickness, or why some people recover fully while others experience long-term effects like post-polio syndrome or long-COVID [15]. This is because we are unable to foresee how an individual’s immune response and immunological-viral interactions would develop. As a result, we cannot currently reliably forecast how a given patient will react to therapy with immune modulators or antiviral medications, whether for endemic circulating viruses like seasonal flu or novel viruses brought on by pandemics. However, it is nearly impossible to anticipate immune system behavior using qualitative models because parameters such as: cytokine levels and immune cell profiles can change in complex ways over time and geographically [16].

The complexity of viral infection and immune response has led to the development of mechanistic computational models, which differ in the mathematical and computational representation of components, interactions, levels of spatial detail, and the time scales they consider [17]. The model of infection is based on scientifically driven theories that identify the essential physical elements of immune response and viral infection and how these elements interact to produce infection (considering essential measurable and quantifiable variables to best capture these elements and interactions). Moreover, it is important to know how to translate the dynamic mechanistic model into a computer simulation to construct a mechanistic computational model [4,18]. For instance, we must decide whether to characterize individual virions or viral concentrations, or whether changes occur continuously over time or as stochastic events. Finally, we must consider that there are time limits on the windows for successful therapies, and some treatments might only be effective when used as prophylactic measures or in the early stages of infection [19].

### Interplay of Viral Infection Dynamics and the Immune System on Modelling

The development of mathematical immunology modelling has been influenced by the complexity of understanding the immune response to HIV infection. The target-cell-limited model, initially, proposed to understand the dynamics of HIV infection provided a foundation for within-host modelling [20]. This framework was afterwards expanded in numerous ways and applied to other viral illnesses. Three factors make up the straightforward target-cell-limited model: the viral load, infected virus-producing cells, and cells that are sensitive yet not infected. The lifespans of infected target cells and virus particles could be estimated by fitting this model to the viral-load data [21]. It also permitted assessment of the pace at which infected cells produce virions. These early models laid the foundation for the subsequent creation of far better HIV medicines.

For example, the innate immune system’s first line of defense is the interferon (IFN) family of proteins, which block viral replication inside infected cells and stop host cells from becoming infected. The importance of innate immune response feedback loops in influencing how diseases develop in people has been discussed in recent models [22]. Moreover, the simple target-cell-limited model could not explain observed primary HIV dynamics in an infected host after the initial acute viral-load peak, raising the possibility that cytotoxic T lymphocytes (CTLs) and cytokine-suppressive viral replication may be involved in regulating viral load [20].

Insights with significant therapeutic utility have been revealed by models that incorporate the adaptive immune response. For instance, the “post-treatment control” of HIV viral load observed in some HIV patients was explained by an effector-cell response and exhaustion model [23], and it was suggested that therapeutic vaccination to boost effector-cell response before stopping antiretroviral therapy might increase the likelihood of post-treatment viral load control. Models were developed to optimize the duration of the therapy and minimize cost and toxicity of direct-acting antiviral (DAA) medication. Viral-kinetic models can forecast the length of DAA therapy required to reach a cure in patients infected with HCV and thus tailor the treatment [23]. These models can be applied to early viral-kinetic data under drug treatment. Besides the model could suggest the one patient who relapsed would have benefited from taking sofosbuvir + ledipasvir for an additional week [24]. By assuming that HCV RNA in serum contains both infectious and non-infectious viruses, Goyal et al. expanded these models [25], which helps to explain why some people can be cured by ultrashort DAA therapy.

According to Baral et al., the viral reduction brought on by DAAs during chronic HCV treatment may have prevented CTL depletion, allowing the virus to be eliminated after treatment. The model was able to estimate the required length of DAA therapy for each patient and, as a result, tailor the treatment by defining a good response for specific patients [26].

The length of infection Influences both the rate at which viral particles are produced and the mortality rate of infected cells and age-structured models with detailed submodels of the viral life cycle can be used for the systematic exploration of new drug targets [14,27]. All viruses replicate in the same way: they establish contact with a target cell in the host, release genetic material into the cell, use the machinery of the cell to replicate, assemble new viral particles, and release those particles from the infected cell [28]. Which of these steps should be blocked for the quickest and most efficient therapies can be determined by mechanistic models? Models, for instance, can investigate how the number of infections impacts the viral replication rate. Model simulations can also predict the impact of drug-based perturbations when viral replication pathways involve both positive and negative feedback [29].

We may anticipate that the best course of action in situations when we have a highly powerful antiviral would be to seek treatment as soon as possible following either infection or diagnosis. Delaying antiviral medication increases the danger of immunological reactions and virus-induced tissue damage. Early antiviral therapy, however, may prevent the adaptive immune response from being activated and, as a result, the development of long-term protective immunity [30]. This adjustment was studied in a model by Stromberg et al., who suggested a short window following infection during which antiviral therapy could lessen disease symptoms without obstructing the development of long-term immunity, ensuring that those infected receive the benefits of vaccination with a lower risk of the disease [31].

The immune system’s cells react to a wide range of signals, many of which arrive at the same time. We can investigate the biochemical mechanisms underlying such reactions with the aid of computational models using signaling pathways and cellular behavior, particularly when those models strive to include molecular specifics of intracellular reaction networks [15,32]. Such detailed models may focus on the interactions between molecule binding domains and how these interactions are influenced by elements such as post-translational modifications or steric restraints in multi-molecular complexes. The mechanistic immunological hypotheses can then be formalized and tested through quantitative simulations [33]. However, we need to be aware that comprehension of multi-signal cellular responses and their interaction at the tissue level is the most difficult challenge we face in our quest to understand immune cell function because it can be too reductionist [34]. Learning how to employ drugs to understand, strengthen and replicate immune system function may be preferable to trying to simulate all the intricacies mentioned above.

Table 2 shows that there are a number of mathematical models that have been developed to understand the dynamics of HIV, Influenz and SARS-CoV-2 infection and the effects of antiretroviral therapy (ART) on the different diseases. These models typically involve the use of differential equations to describe the interations between the virus and the immune system, as well as the effects of ART on the virus. These types of models can be used to simulate the course of infection and the effects of different ART regimens on the virus, and can provide insights into how to optimize treatment strategies. However, it’s important to note that these models are simplified representations of complex biological systems, and their predictions may not always be accurate in real-world situations.

Ideally, if patients were treated based on the knowledge obtained from their immune status, drug response, and predictive models, therapy failure would be minimized, and severe diseases could be better controlled. This would also improve our ability to forecast how a given patient would react to the therapy. It is indeed true that virus infections can be complex and can affect different tissues or organs, and that the choice of treatment can depend on a number of factors, including the specific virus, the patient’s immune status, and the severity of the infection.

One approach to developing a patient-specific antiviral therapy strategy is to use a mathematical model that takes into account a panel of patient-specific parameters that may be related to the virus or the host immune response. This type of model could be used to forecast drug efficacy or to guide the selection of drugs for a particular patient.

There are a number of different approaches that could be used to build such a model, depending on the specific goals of the treatment and the data available. For example, the model could be based on data from clinical trials or observational studies, or it could incorporate data on the patient’s specific genetic and immunologic characteristics.

It’s important to note that developing a patient-specific antiviral therapy strategy based on a mathematical model is still a complex and challenging task, and more research is needed to fully understand the factors that influence the effectiveness of different antiviral therapies. However, by using quantitative approaches like this, it may be possible to optimize treatment choices and improve outcomes for patients with virus infections.

## 4. Strengthening the Immune System

Numerous infections alter the immune response, some of those modifications are time dependent, and can lead to T-cell exhaustion. A strategy to deal with these phenomena is to use immune-boosting drugs, since they are essential to track and treat viral infections [41]. The symptoms of viruses like HIV or HBV that can take months to appear, when detected these infections are regarded as chronic. On the other side, acute infections like the flu begin to manifest symptoms earlier, two days after exposure, and most patients recover from the infection within seven days. For example, COVID-19 manifests itself 5 to 7 days after exposure (however, for some people, the illness can last for weeks or even months) [42].

Intracellular production of IL-2, IFN-g, and TNF, and often severely reduced in persistent viral infections, and T cells also express large numbers of inhibitory receptors, including PD-1 (programmed cell death-1) and Lymphocyte-Activation Gene 3 (Lag-3). These phenotypic alterations exhaust the functional T cell response, as does the immune system’s inability to eliminate pathogens in chronic infection [43]. These largely encourage terminally differentiated T cells while preventing CD8+ T cell memory development. Additionally, when PD-1 signalling is suppressed, CD8+ T responsiveness and antiviral activity markedly increase. These results indicate a permanent cessation of the T cell immunological response. However, the molecular mechanisms underpinning the maintenance of terminally differentiated T cells in persistent infections and the enhancement in T cell responsiveness following checkpoint inhibition are still poorly understood [44].

Immune-activating strategies were the turning point to reduce cytokine storms. In addition to targeting T cells, therapies that regulate pro-inflammatory factors, such as cytokines, can also be used in the treatment of ARDS caused by respiratory viruses. Examples of therapies that target pro-inflammatory cytokines are TNF inhibitors and interleukin inhibitors. These therapies work by inhibiting the production or activity of specific cytokines, which can help to reduce inflammation and improve outcomes in patients with ARDS. It’s important to note that these therapies are typically used in combination with other treatments, such as oxygen therapy, mechanical ventilation, and supportive care, to manage the symptoms of ARDS [41]. However, conclusive evidence of their usefulness is still not available, although IL-7 and anti-PD1/PDL1 therapies, have showed early signs of dices in early clinical trials.

Moreover, numerous in vitro and in vivo studies reveal that different viruses can cause a metabolic state like Warburg’s. These pathogens manipulate the metabolism of the host cell to divert glycolysis and TCA toward the manufacture of amino acids, lipids, and nucleotides, which is necessary for their survival and sustenance [45]. After viral infection, the number of several glycolytic pathway intermediaries is considerably enhanced. Some viruses cause metabolic changes to balance the ROS generated by the host during the infection response or to aid in the reproduction of the virus [46]. Oncogenic viruses can cause a Warburg-like effect or altered metabolic status, including the human HPV, HBV and HCV, EBV, among others [47]. Cellular metabolism affects immune cell activation, the release of cytokines, and antitumor or antiviral activity. As a result of viral infection, cells frequently experience increased glucose metabolism (leading to aerobic glycolysis, or the so-called Warburg effect), and their lipid metabolism typically shifts from fatty acid oxidation (FAO) to fatty acid synthesis (FAS). Particularly for those viruses that are encapsulated, more FAS is required. Only metabolically active cells are infected by some viruses, and the infection frequently serves to downregulate one or more metabolic activities [46].

In clinical research, it is usual to combine new metabolic modulators with immune checkpoint inhibitors because it appears that anti-PD1/PD-L1 antibodies may help T cells regain their metabolic fitness [48]. A widespread testing of combination immunometabolism strategies has been made possible by immune checkpoint inhibitors’ good safety profile (especially against PD1 and PD-L1). However, the combination of these drugs is not always beneficial or effective. This highlights the fact that this type of interventional target is not easily modulated; they represent a significant challenge requiring carefully calibrated approaches, preferably based on reliable biomarkers and new technical strategies specifically targeting TME [49].

### 4.1. Reverse T Cell Exhaustion

**Figure 1:**
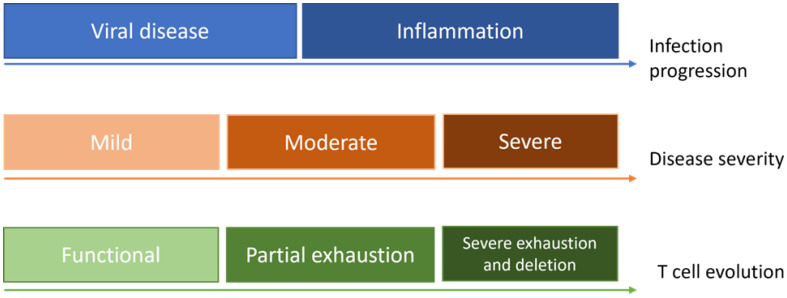
Simplistic demonstration of T cell evolution according to infection progression and disease severity.

T cell fatigue is a condition of T cell malfunction that develops following numerous long-term infections and malignancies (Figure 1). It’s transcriptional state is different from that of functional effector or memory T cells, having extended expression of inhibitory receptors, and low effector activity. The appropriate course of treatment for each patient is determined by using functional assays to count the number of activated T cells in the patient blood samples. This phenomenon is reported in cancer and infections such as HBV, HCV, and HIV [44,50].

Therapeutic immunological checkpoints for T-cell fatigue are co-inhibitory receptors, this act during acute infections to dampen immune responses, which are down-regulated once the pathogen is eradicated to restore homeostasis and memory T cell development (Table 3) [51]. However, this arrangement changes with chronic infections, when co-inhibitory receptors are more highly and persistently expressed [52]. The expression patterns, ligands, and signaling motifs of co-inhibitory receptors differ, and the molecular processes by which they regulate T-cell exhaustion are still poorly understood. However, the identification of their significance in the dysregulation of cellular immune responses in infected hosts has provided fresh opportunities for the development of therapeutic molecules to reestablish the optimum immune response.

Due to its upregulation of these cells and decreased T cell function, the receptor PD1 is a crucial indicator of T cell exhaustion. By reactivating the anticancer immune response, antibodies that target PD1, its ligand (PDL1), or CTLA4, another inhibitory surface receptor, have changed cancer therapy. But the LCMV model was where PD1-targeting antibodies first showed promise (an important model for studying viral pathogenesis and immune responses). In HIV-infected individuals, PD1-blocking antibodies showed increased T cell counts and function. [53]. PD-1 expression on HIV-specific CD8+ T cells is positively correlated with elevated viral load, decreased CD8+ T cell activity, disease progression, and decreased CD4+ T cell counts during HIV-1 infection. Antiretroviral therapy (ART) can reduce the expression of PD-1 on virus-specific T lymphocytes in HIV-infected patients. Long-term non-progressors (LTNPs) have lower levels of PD-1 expression on virus-specific T cells, therefore these populations have multiple functions than T cells in progressors. [54]. By increasing the proliferative potential of T cells and stimulating cytokine production, in vitro studies demonstrate that inhibiting the PD-1 pathway improves pathogen control and restores T-cell functionality.

More recently, SIV infection therapy with PD-1 inhibitory antibody has boosted the frequencies and functional quality of SIV-specific CD8+ T lymphocytes detectable in the blood and gut, viral loads have dropped, and survival rates in infected macaques have increased dramatically [55].

The changing aspects and importance of the PD-1 pathway have also been studied in HBV and HCV infections, in addition to HIV. HCV-specific CD4+ and CD8+ T cell responses were associated with reducing viral growth [56]. Curiously, PD-1 expression on HCV-specific CD8+ T cells increased markedly in the liver, even though blocking PD-1 had no beneficial effect on the activities of these cells. This demonstrates that a variety of factors must interact and regulate the maintenance of T-cell exhaustion and suggests that the distribution and concentration of viral antigen as well as the compartmentalization of the virus-specific T cells have a significant impact on the degree of exhaustion [57].

Moreover, the metabolism of active T cells and fatigued T cells differs. Redox stress in T cells is caused by long-term viral infections or tumours, and it results in cell death and lymphodepletion. In persistent LCMV infection, early effector CD8+ T lymphocytes’ glycolytic and mitochondrial metabolism are inhibited by PD-1 signalling [58]. A critical metabolic regulator known as peroxisome proliferator-activated receptor gamma coactivator 1 (PGC1-alpha) was shown to be blocked by PD-1 signals. Interestingly, overexpressing PGC1-alpha corrected some metabolic abnormalities in developing worn-out T cells and improved effector function. [59]. Naive and resting T cells use fatty acid oxidation (FAO) and the mitochondrial tricarboxylic acid (TCA) cycle to produce a sizable quantity of ATP through oxidative phosphorylation (OXPHOS).

Furthermore, recent studies employing a mouse model demonstrated that mitochondrial activity was a need for the generation and maintenance of antigen-specific responses. T cells triggered in this way change their metabolism to high rates of glycolysis even in the presence of ample oxygen, supporting proliferation and effector function by supplying fast energy and metabolites [46].

For example, OXPHOS was elevated by HIV-specific T lymphocytes since there was more mitochondria. However, whether the growth in the number of functional mitochondria resulted from an increase in the number of functional mitochondria or from the formation of a considerable number of non-functional mitochondria is uncertain [60,61]. Plus, a constitutive glucose transporter called GLUT1 is upregulated by the poorly functioning PD-1hi T cell responses against HBV, according to recent research by Schurich et al. [62]. They also demonstrated that, in contrast to the more capable CMV-specific T cells, which were able to use OXPHOS in the absence of glucose, Glut1hi HBV-specific T cells are dependent on glucose supply. In addition to being unable to convert to OXPHOS, the tired HBV-specific T cells also had larger mitochondria and decreased mitochondrial potential, all of which are signs of mitochondrial malfunction [62].

The antiviral activity of worn-out HBV-specific CD8+ T cells is markedly improved by mitochondrion-targeted antioxidants, indicating the crucial role of ROS in T-cell fatigue [63]. This findings show how we can used drugs to infer on the immune system dynamics, and the crucial necessity to carry out further studies on (1) how mitochondrial biogenesis influences T-cell exhaustion, and (2) how to treat chronic viral infection by focusing on the mitochondrial metabolism of T cells. Additionally, epigenetic mechanisms directly control the transcription of T cell exhaustion [64].

### 4.2. Measurement of Exhaustion and Prediction of the Drug Response by T Cell Subtype Profiling

The depletion of T cells impairs the antiviral T cells’ capacity to clear viruses from the body. Exhausted T cells differ from usual effector and memory subsets. Five T cell subtypes—naïve, activated, effector memory (EM), central memory (CM), and exhausted—can be distinguished based on the activation and differentiation status of T cells [65]. These subtypes describe the immunogenic status of the T cell adaptive immune response and, when measured separately, have provided insights into the response to anti-PD-1 therapy [66,67].

By profiling the different T cell subtypes, we would be able to predict the immune response and forecast how a given patient will react to therapy with immune modulators or antiviral medication [68]. In a recent paper, T Cell Subtype Profiling (TCSP), a technique developed by Schillebeeckx et al., uses five RNA models to describe the prevalence of T cell subtypes (TCSs) in FFPE tissues. This method is analytically validated and corroborates associations between TCSs and clinical outcomes. Based on TCS estimates it was possible to predict response to anti-PD-1 therapy in three different cancers and outperform the indicated PD-L1 test, as well as Tumor Mutational Burden [69]. This model of exhaustion has its origins in viral research and the authors investigated viral and tumour-induced exhaustion in tandem, by performing TCSP on solid tumours with etiologies involving persistent viral infection. Regardless of viral state, findings showed that fatigued T cells are increased in at least some tumour types with concomitant viral infection, but this may vary depending on the virus. TCS profiles were similar in both malignant and non-malignant tissue. However, TCSP was able to detect elevated levels of weariness brought on by both viral and tumor-induced exhaustion.

Understanding the patient’s T cell profile and the degree of exhaustion of T cells, treatment can be guided by those profiles to mediate viral infection and immune response. For example, Davis et al. concluded that ibrutinib has a protective effect on T cells in patients with chronic lymphocytic leukaemia [70]. Therefore, short-term ibrutinib treatments could be helpful to mediate immune response during viral infections. PD-1 plays a crucial role in regulating T-cell exhaustion by increasing T-cell proliferative potential and fostering cytokine production. Inhibiting the PD-1 pathway promotes pathogen control while restoring T-cell functionality [44]. Cytokines can also reinvigorate exhausted T cells. The persistence of viral infections like HCV, HIV, and EBV has been observed to be positively correlated with IL-10, suggesting that viruses may employ this association to avoid host immune systems. Blocking IL-10 in LCMV infection models seems to reduce viral persistence and improve T-cell performance. In HIV infection, IL-10 blockage is also used, and it causes a markedly enhanced release of IFN- by CD4+ T cells. However, combining IL-10 blockage with PD-1 blocking only allows for the restoration of a small subset of T cell cytokines, such as IL-2 [71,72].

Moreover, enhanced T Cell metabolism has its indicators. Increased numbers of memory T cell subsets, enhanced cell proliferation, and decreased exhaustion indicators were seen when glycolysis was inhibited when cultivating T cells, indicating that glycolysis may be a significant component affecting T cell metabolism [73]. The transcriptional coactivator can be inhibited by the interaction between PD-L1 and PD-1, which can also control early glycolysis levels and mitochondrial alterations. By lowering aspartic acid consumption, Metformin, an antidiabetic agent, seems to have extra valuable actions, even on viral infections. Changes in metabolic reprogramming, which facilitates T-cell reinvigoration, are also brought about by anti-PD-1 inhibition. In contrast, anti-CTLA4 mABs cause the genes involved in the cell cycle and proliferation to become less active. Pre-clinical research demonstrated that CTLA4 inhibition causes Treg numbers to decrease, which increases the effectiveness of treatment against tumours. Additionally, anti-CTLA4 therapies increase functional reactivity by altering the TCR repertoire [73].

## 5. Conclusions

Antiretroviral drug administration, the prevalence of drug resistance, the overall degree of viral generation, and the potency of immune responses are all dynamically correlated. Viral and immunological processes interact in extremely complicated and non-linear ways. Mathematical models can help characterize these non-linear interactions, in addition to providing reliable analysis and quantitative forecasts. They can be used to follow the immune system and create a profile of disease for each patient. Since SARS-CoV-2 outbreak a huge number of models were made public, and it was noticeable the effort to model viral infection in conjunction with the immune systems. Although mechanistic computational models have a tremendous potential to advance the research and application of antiviral medicines, their development and utilization requires we continue to work to achieve new levels of knowledge. To increase our abilities to treat patients and identify the individual differences in disease development, immune response profiling might be useful. But if researchers and modelers don’t pose the correct questions, the solutions provided by the models will address the wrong issues. Moreover, the information retrieved from pharmacological use is also essential. The drugs interact with each individual, at the different stages of viral proliferation and immune status can be elucidating in how to deal with T cell fatigue, for example. This information in conjunction with mathematical models can be quite beneficial and complementary. Therefore, efforts to embrace a multidisciplinary approach to solve these issues are fundamental for use to advance. We need, create teams of researchers with different backgrounds in science, and take advantage of the momentum created by SARS-CoV-2 pandemic to connect and answer these questions. Building models and information for the future.

## Figures and Tables

**Table 1:** Different types of antiviral drugs and treatments for viral infections.

Type	Antiviral Drug	Therapeutic Indication	Mechanism of Action
Direct acting antiviral	Convalescent plasma	Commonly used to treat viral infections such as influenza and COVID-19	tHe exact mechanisms of action of convalescent plasma will depend on the specific virus and the specific immune responses that are being targeted. It works by: Neutralizing the virus Enhancing the immune response Modulating the immune response
	Nucleoside analogs	Acyclovir: Used to treat herpes simplex virus infections, including genital herpes and cold sores.	It is converted into its active form, acyclovir triphosphate, inside infected cells. Once inside the cells, acyclovir triphosphate inhibits the activity of a viral enzyme called herpesvirus-specific DNA polymerase, which is responsible for replicating the herpesvirus’s DNA. By inhibiting this enzyme, acyclovir helps to prevent the virus from replicating and spreading to other cells.
		Lamivudine: Used to treat HIV and hepatitis B virus infections.	It is converted into its active form, lamivudine triphosphate, inside infected cells. Once inside the cells, lamivudine triphosphate inhibits the activity of a viral enzyme called reverse transcriptase, which is responsible for replicating the HIV virus’s RNA into DNA. By inhibiting this enzyme, lamivudine helps to prevent the virus from replicating and spreading to other cells.
		Tenofovir: Used to treat HIV and hepatitis B virus infections.	It is converted into its active form, tenofovir diphosphate, inside infected cells. Once inside the cells, tenofovir diphosphate inhibits the activity of a viral enzyme called reverse transcriptase, which is responsible for replicating the HIV virus’s RNA into DNA. By inhibiting this enzyme, tenofovir helps to prevent the virus from replicating and spreading to other cells.
		Valacyclovir: Used to treat herpes simplex virus infections, including genital herpes and cold sores.	It is converted into its active form, tenofovir diphosphate, inside infected cells. Once inside the cells, tenofovir diphosphate inhibits the activity of a viral enzyme called reverse transcriptase, which is responsible for replicating the HIV virus’s RNA into DNA. By inhibiting this enzyme, tenofovir helps to prevent the virus from replicating and spreading to other cells.
		Zidovudine: Used to treat HIV infections	It is converted into its active form, zidovudine triphosphate, inside infected cells. Once inside the cells, zidovudine triphosphate inhibits the activity of a viral enzyme called reverse transcriptase, which is responsible for replicating the HIV virus’s RNA into DNA. By inhibiting this enzyme, zidovudine helps to prevent the virus from replicating and spreading to other cells.
	Monoclonal antibodies	Palivizumab: Used to prevent respiratory syncytial virus (RSV) infections in high-risk individuals, such as premature infants and individuals with compromised immune systems.	It is directed against a specific protein called the F protein, which is found on the surface of the respiratory syncytial virus (RSV). Palivizumab binds to the F protein and prevents the virus from attaching to and infecting cells in the respiratory tract. This helps to prevent the development of RSV infection and its associated complications.
		Remdesivir: Used to treat COVID-19.	It is converted into its active form, remdesivir triphosphate, inside infected cells. Once inside the cells, remdesivir triphosphate inhibits the activity of a viral enzyme called RNA-dependent RNA polymerase, which is responsible for replicating the viral RNA. By inhibiting this enzyme, remdesivir helps to prevent the virus from replicating and spreading to other cells.
		Casirivimab/imdevimab: Used to treat COVID-19.	Are monoclonal antibodies that are directed against a specific protein called the spike protein, which is found on the surface of SARS-CoV-2, the virus that causes COVID-19. Casirivimab and imdevimab bind to the spike protein and prevent the virus from attaching to and infecting cells in the respiratory tract. This helps to prevent the development of COVID-19 and its associated complications.
		Peginterferon lambda: Used to treat hepatitis C virus infection.	Interferons are proteins that are produced by the body’s immune system in response to viral infections. They help to stimulate the immune system to fight the virus and also inhibit the replication of the virus. Peginterferon lambda is a long-acting form of interferon that is given by injection once a week. It is thought to work by stimulating the production of proteins that inhibit the replication of HCV and by activating immune cells to attack the virus.
	Fusion inhibitors	Enfuvirtide: Used to treat HIV infections	It works by binding to a specific protein called gp41, which is found on the surface of the HIV virus and is involved in the process of viral fusion. By binding to gp41, enfuvirtide helps to prevent the virus from fusing with and infecting host cells.
		Maraviroc: Used to treat HIV infections	It works by binding to a specific protein called CCR5, which is found on the surface of host cells and is used by the HIV virus to enter cells. By binding to CCR5, maraviroc helps to prevent the virus from fusing with and infecting host cells.
	Polymerase inhibitors	Ribavirin: Used to treat hepatitis C virus infections and certain types of respiratory virus infections.	It works by inhibiting the activity of viral polymerases, which are enzymes that the virus uses to replicate its genetic material. Ribavirin is thought to work by inhibiting the synthesis of the virus’s genetic material, which helps to prevent the virus from replicating and spreading to other cells.
		Oseltamivir: Used to treat influenza virus infections.	Is a prodrug, meaning that it is converted into its active form, oseltamivir carboxylate, inside infected cells. Once inside the cells, oseltamivir carboxylate inhibits the activity of a viral enzyme called neuraminidase, which is responsible for releasing newly formed viruses from infected cells. By inhibiting this enzyme, oseltamivir helps to prevent the virus from spreading to other cells.
		Zanamivir: Used to treat influenza virus infections.	It works by inhibiting the activity of a viral enzyme called neuraminidase, which is responsible for releasing newly formed viruses from infected cells. By inhibiting this enzyme, zanamivir helps to prevent the virus from spreading to other cells.
		Sofosbuvir: Used to treat hepatitis C virus infections.	It works by inhibiting the activity of a viral enzyme called NS5B polymerase, which is responsible for replicating the HCV virus’s RNA. By inhibiting this enzyme, sofosbuvir helps to prevent the virus from replicating and spreading to other cells.
	Receptor decoys	Maraviroc: Used to treat HIV infections	The mechanisms of this drugs are described above. Receptor decoys and fusion inhibitors are similar in that they both work by blocking the interaction between a virus and its target cell. Receptor decoys are a class of direct-acting antiviral drugs that work by blocking the interaction between a virus and its target cell. They do this by providing a “decoy” receptor that the virus can bind to, which prevents the virus from attaching to and infecting the target cell. Fusion inhibitors are a class of direct-acting antiviral drugs that work by inhibiting the process of viral fusion
		Enfuvirtide: Used to treat HIV infections	
Host-factor antiviral	Endocytosis inhibitors	Amantadine: Used to treat influenza virus infections	It works by inhibiting the activity of a viral enzyme called M2, which is responsible for releasing newly formed viruses from infected cells. By inhibiting this enzyme, amantadine helps to prevent the virus from spreading to other cells.
		Rimantadine: Used to treat influenza virus infections	
		Oseltamivir: Used to treat influenza virus infections	Oseltamivir is a prodrug, meaning that it is converted into its active form, oseltamivir carboxylate, inside infected cells. Once inside the cells, oseltamivir carboxylate inhibits the activity of a viral enzyme called neuraminidase, which is responsible for releasing newly formed viruses from infected cells. By inhibiting this enzyme, oseltamivir helps to prevent the virus from spreading to other cells.
	Interferons	Alpha interferon (IFN-α) is produced by leukocytes (white blood cells) and is effective against a wide range of viruses, including herpesvirus, hepatitis B and C viruses, and human papillomavirus (HPV).	Interferons (IFN) work by activating interferon receptors on the surface of infected cells, which triggers a signaling pathway that leads to the production of proteins called antiviral effectors. These effectors inhibit the replication of viruses by blocking the production of viral proteins, disrupting viral assembly and release, and activating the immune system to clear the infection.
		Beta interferon (IFN-β) is produced by fibroblasts (a type of cell found in connective tissue) and is effective against certain viruses, including herpes simplex virus and HIV.	
		Gamma interfero (IFN-γ) is produced by immune cells called T cells and natural killer cells, and is effective against a variety of viruses, including HIV and hepatitis C virus.	
	Kinase inhibitors	Maraviroc is an HIV-1 entry inhibitor	Targets the CCR5 co-receptor on immune cells. It blocks the interaction between HIV-1 and CCR5, preventing the virus from entering the cell.
		Selzentry (maraviroc) is an HIV-1 entry inhibitor	Targets the CCR5 co-receptor on immune cells. It blocks the interaction between HIV-1 and CCR5, preventing the virus from entering the cell.
		Acyclovir is an antiviral drug that is effective against herpesvirus infections.	It works by inhibiting the viral enzyme thymidine kinase, which is required for the synthesis of viral DNA
		Ganciclovir is an antiviral drug that is effective against cytomegalovirus (CMV) infections.	It works by inhibiting the viral enzyme DNA polymerase, which is required for the synthesis of viral DNA
		Valganciclovir is a prodrug of ganciclovir that is used to treat CMV infections.	It is converted to ganciclovir by the enzyme valacyclovir hydrolyase, which is expressed in infected cells
		Raltegravir is an HIV-1 integrase inhibitor	Blocks the integration of HIV-1 DNA into the host genome. It works by inhibiting the activity of the HIV-1 integrase enzyme
	Lipidomic drugs	Amantadine is an antiviral drug that is effective against influenza A virus.	It works by blocking the ion channel M2, which is required for the release of viral particles from infected cells.
		Oseltamivir is an antiviral drug that is effective against influenza A and B viruses.	It works by inhibiting the viral enzyme neuraminidase, which is required for the release of viral particles from infected cells.
		Zanamivir is an antiviral drug that is effective against influenza A and B viruses.	It works by inhibiting the viral enzyme neuraminidase, which is required for the release of viral particles from infected cells.
		Remdesivir is an antiviral drug that is effective against a wide range of RNA viruses, including SARS-CoV-2 (the virus that causes COVID-19).	It works by inhibiting the viral RNA polymerase enzyme, which is required for the synthesis of viral RNA.
		Favipiravir is an antiviral drug that is effective against RNA viruses including influenza A and B viruses and Ebola virus.	It works by inhibiting the viral RNA polymerase enzyme, which is required for the synthesis of viral RNA.
		Sofosbuvir is an antiviral drug that is effective against hepatitis C virus (HCV).	It works by inhibiting the viral NS5B RNA polymerase enzyme, which is required for the synthesis of viral RNA.
Direct-acting and host-factor antiviral	Protease inhibitors	Saquinavir is an HIV protease inhibitor	Blocks the protease enzyme, which is required for the processing of viral proteins.
		Ritonavir is an HIV protease inhibitor	
		Nelfinavir is an HIV protease inhibitor	
		Lopinavir is an HIV protease inhibitor	
		Boceprevir is an HCV protease inhibitor	
		Telaprevir is an HCV protease inhibitor	
		Camostat mesilate is an antiviral drug that is effective against a variety of viruses, including influenza A and B viruses, respiratory syncytial virus, and norovirus.	It works by inhibiting the protease enzyme trypsin, which is required for the release of viral particles from infected cells.
		Fosamprenavir is an HIV protease inhibitor that is converted to its active form, amprenavir, by the enzyme CYP3A4.	It blocks the protease enzyme, which is required for the processing of viral proteins
		Indinavir is an HIV protease inhibitor	That blocks the protease enzyme, which is required for the processing of viral proteins
		Nelfinavir is an HIV protease inhibitor	
		Ritonavir is an HIV protease inhibitor	
		Saquinavir is an HIV protease inhibitor	
	Translation inhibitors	Efavirenz is an HIV reverse transcriptase inhibitor that blocks the synthesis of viral DNA from viral RNA.	Blocks the synthesis of viral DNA from viral RNA. It works by inhibiting the activity of the HIV reverse transcriptase enzyme, which is required for the synthesis of viral DNA from viral RNA.
		Emtricitabine is an HIV reverse transcriptase inhibitor	Blocks the synthesis of viral DNA from viral RNA. It works by inhibiting the activity of the HIV reverse transcriptase enzyme, which is required for the synthesis of viral DNA from viral RNA.
		Tenofovir is an HIV reverse transcriptase inhibitor that blocks the synthesis of viral DNA from viral RNA.	It works by inhibiting the activity of the HIV reverse transcriptase enzyme, which is required for the synthesis of viral DNA from viral RNA.
		Zidovudine is an HIV reverse transcriptase inhibitor	It works by inhibiting the activity of the HIV reverse transcriptase enzyme, which is required for the synthesis of viral DNA from viral RNA.
		Baricitinib is an antiviral drug that is effective against a variety of RNA viruses, including influenza A and B viruses, respiratory syncytial virus, and norovirus.	It works by inhibiting the translation of viral mrna into viral proteins. It does this by inhibiting the activity of the ribosomes, which are the cellular machinery that reads the genetic code in mrna and synthesizes proteins based on that code.
		Ruxolitinib is an antiviral drug that is effective against a variety of RNA viruses, including influenza A and B viruses, respiratory syncytial virus, and norovirus.	It works by inhibiting the translation of viral mrna into viral proteins. It does this by inhibiting the activity of the ribosomes, which are the cellular machinery that reads the genetic code in mrna

**Table 2:** Types of the most severe and prevalent diseases caused by virus infections that can be cured by modulating the immune response.

Disease	How It Can Be Modulated?
Acquired immunodeficiency syndrome (AIDS), which is caused HIV	Example 1: HIV-1 immune dynamics model, which was developed by Nowak and May in 1996 [35]. This model describes the interactions between HIV, CD4+ T cells, and antiviral drugs. The model includes equations that describe the rate of HIV replication, the rate of CD4+ T cell production and loss, and the rate at which antiviral drugs inhibit HIV replication. Example 2: HIV-1 treatment intensification model, which was developed by Guedj et al. in 2007 [36]. This model is based on the HIV-1 immune dynamics model and incorporates additional equations to describe the effects of different ART regimens on the virus.
Influenza caused by the influenza virus	Example 1: influenza transmission model, which was developed by Ferguson et al. in 2005 [37]. This model describes the transmission of influenza from person to person, as well as the effect of antiviral drugs on the virus. The model includes equations that describe the rate of influenza transmission, the rate of antiviral drug-induced reduction in the number of infectious individuals, and the rate at which individuals become immune to the virus. Example 2: influenza vaccine effectiveness model, which was developed by Thompson et al. in 2006 [38]. This model is based on the influenza transmission model and incorporates additional equations to describe the effects of vaccination on the virus and the immune system. The model is based on the influenza transmission model, which was developed by Ferguson et al. in 2005, and incorporates additional equations to describe the effects of vaccination on the virus and the immune system. It’s important to note that the model is based on a specific set of assumptions and may not be applicable to all situations
Severe acute respiratory syndrome (SARS), which is caused by the SARS-CoV-2 virus	Example 1: SEIR (susceptible-exposed-infectious-recovered) model, which is a type of compartmental model that describes the transmission of infectious diseases. The SEIR model includes equations that describe the rate of transmission of SARS-CoV-2, the rate of recovery, and the rate at which individuals become immune to the virus [39]. Example 2: SIR (susceptible-infectious-recovered) model, which is a variant of the SEIR model that does not include an exposed compartment. The SIR model has been used to study the effectiveness of various interventions, such as antiviral drugs and vaccines, on the transmission of SARS-CoV-2 [40].

**Table 3:** Treatment approach and its targets to reverse T-cell exhaustion.

Treatment Approach	Target
Antibody blockade	PD-1/PD-L1
Altering metabolic pathways	PCK1 GLS1 Pyruvate kinase
Decrease PD-1 expression	FoxOT TOX
Improve TCR signaling	AKT IL-10 or IL10R IL.2R

## Data Availability

Not applicable.

## References

[1] Rai K.R., Shrestha P., Yang B., Chen Y., Liu S., Maarouf M., Chen J.L. (2021). Acute Infection of Viral Pathogens and Their Innate Immune Escape. Front. Microbiol..

[2] Boldogh I., Albrecht T., Porter D.D., Baron S. (1996). Persistent Viral Infections. Medical Microbiology.

[3] Lambert P.F., Sugden B. (2006). Cancer issue: Viruses and Human Cancer. Yale J. Biol. Med..

[4] Smith A.M. (2018). Validated models of immune response to virus infection. Curr. Opin. Syst. Biol..

[5] Zitzmann C., Kaderali L. (2018). Mathematical analysis of viral replication dynamics and antiviral treatment strategies: From basic models to age-based multi-scale modeling. Front. Microbiol..

[6] Zeitlinger M., Koch B.C.P., Bruggemann R., De Cock P., Felton T., Hites M., Le J., Luque S., MacGowan A.P., Marriott D.J.E. (2020). Pharmacokinetics/Pharmacodynamics of Antiviral Agents Used to Treat SARS-CoV-2 and Their Potential Interaction with Drugs and Other Supportive Measures: A Comprehensive Review by the PK/PD of Anti-Infectives Study Group of the European Society of Antimicrobial Agents. Clin. Pharmacokinet..

[7] Kausar S., Khan F.S., Ur Rehman M.I.M., Akram M., Riaz R., Rasool G., Khan A.H., Saleem I., Shamim S., Malik A. (2021). A review: Mechanism of action of antiviral drugs. Int. J. Immunopathol. Pharmacol..

[8] Meganck R.M., Baric R.S. (2021). Developing therapeutic approaches for twenty-first-century emerging infectious viral diseases. Nat. Med..

[9] Maginnis M.S. (2018). Virus–Receptor Interactions: The Key to Cellular Invasion. J. Mol. Biol..

[10] Mahajan S., Choudhary S., Kumar P., Tomar S. (2021). Antiviral strategies targeting host factors and mechanisms obliging +ssRNA viral pathogens. Bioorg. Med. Chem..

[11] Bauer L., Lyoo H., van der Schaar H.M., Strating J.R., van Kuppeveld F.J. (2017). Direct-acting antivirals and host-targeting strategies to combat enterovirus infections. Curr. Opin. Virol..

[12] Hope J.L., Bradley L.M. (2021). Lessons in antiviral immunity. Science.

[13] Almocera A.E.S., Nguyen V.K., Hernandez-Vargas E.A. (2018). Multiscale model within-host and between-host for viral infectious diseases. J. Math. Biol..

[14] Zarnitsyna V.I., Gianlupi J.F., Hagar A., Sego T.J., Glazier J.A. (2021). Advancing therapies for viral infections using mechanistic computational models of the dynamic interplay between the virus and host immune response. Curr. Opin. Virol..

[15] Bellomo N., Bingham R., Chaplain M.A.J., Dosi G., Forni G., Knopoff D.A., Lowengrub J., Twarock R., Virgillito M.E. (2020). A multiscale model of virus pandemic: Heterogeneous interactive entities in a globally connected world. Math. Model. Methods Appl. Sci..

[16] Cicchese J.M., Evans S., Hult C., Joslyn L.R., Wessler T., Millar J.A., Marino S., Cilfone N.A., Mattila J.T., Linderman J.J. (2018). Dynamic balance of pro- and anti-inflammatory signals controls disease and limits pathology. Immunol. Rev..

[17] Waites W., Cavaliere M., Danos V., Datta R., Eggo R.M., Hallett T.B., Manheim D., Panovska-Griffiths J., Russell T.W., Zarnitsyna V.I. (2022). Compositional modelling of immune response and virus transmission dynamics. Philos. Trans. R. Soc. A.

[18] Nguyen L.K.N., Megiddo I., Howick S. (2020). Simulation models for transmission of health care–associated infection: A systematic review. Am J Infect Control..

[19] Handel A., La Gruta N.L. (2019). Thomas PGSimulation modelling for immunologists. Nat. Rev. Immunol..

[20] Perelson A.S. (2013). Ribeiro RMModeling the within-host dynamics of HIV infection. BMC Biol..

[21] Perelson A.S., Ke R. (2021). Mechanistic modelling of SARS-CoV-2 and other infectious diseases and the effects of therapeutics. Clin. Pharmacol. Ther..

[22] Venugopal V., Padmanabhan P., Raja R., Dixit N.M. (2018). Modelling how responsiveness to interferon improves interferon-free treatment of hepatitis C virus infection. PLoS Comput. Biol..

[23] Bolouri H., Young M., Beilke J., Johnson R., Fox B., Huang L., Santini C.C., Hill C.M., Vries A.R.V., Shannon P.T. (2020). Integrative network modeling reveals mechanisms underlying T cell exhaustion. Sci. Rep..

[24] Gorstein E., Martinello M., Churkin A., Dasgupta S., Walsh K., Applegate T.L., Yardeni D., Etzion O., Uprichard S.L., Barash D. (2020). Modeling based response guided therapy in subjects with recent hepatitis C infection. Antivir. Res..

[25] Goyal A., Lurie Y., Meissner E.G., Major M., Sansone N., Uprichardag S.L., Cotler S.J., Dahar H. (2017). Modeling HCV cure after an ultra-short duration of therapy with direct acting agents. Antivir. Res..

[26] Baral S., Roy R., Dixit N.M. (2018). Modeling how reversal of immune exhaustion elicits cure of chronic hepatitis C after the end of treatment with direct-acting antiviral agents. Immunol. Cell Biol..

[27] Nelson P.W., Gilchrist M.A., Coombs D., Hyman J.M., Perelson A.S. (2004). An age-structured model of hiv infection that allows for variations in the production rate of viral particles and the death rate of productively infected cells. Math. Biosci. Eng..

[28] Rampersad S., Tennant P. (2018). Replication and Expression Strategies of Viruses. Viruses: Molecular Biology, Host Interactions and Applications to Biotechnology.

[29] Kapitanov G.I., Chabot J.R., Narula J., Roy M., Neubert H., Palandra J., Farroki V., Johnson J.S., Webster R., Jones H.M. (2021). A Mechanistic Site-Of-Action Model: A Tool for Informing Right Target, Right Compound, and Right Dose for Therapeutic Antagonistic Antibody Programs. Front. Bioinform..

[30] Weber F. (2021). Antiviral Innate Immunity: Introduction. Encycl. Virol..

[31] Stromberg S.P., Antia R. (2011). Vaccination by Delayed Treatment of Infection. Vaccine.

[32] Germain R.N., Meier-Schellersheim M., Nita-Lazar A., Fraser I.D. (2011). Systems biology in immunology: A computational modeling perspective. Annu Rev Immunol..

[33] Culos A., Tsai A.S., Stanley N., Becker M., Ghaemi M.S., McIlwain D.R., Fallahzadeh R., Tanada A., Nassar H., Espinosa C. (2020). Integration of mechanistic immunological knowledge into a machine learning pipeline improves predictions. Nat. Mach. Intell..

[34] Basson M.A. (2012). Signaling in cell differentiation and morphogenesis. Cold Spring Harb. Perspect. Biol..

[35] Nowak M.A., Bangham C.R. (1996). Population dynamics of immune responses to persistent viruses. Science.

[36] Guedj J., Thiébaut R., Commenges D. (2007). Maximum likelihood estimation in dynamical models of HIV. Biometrics.

[37] Ferguson N., Cummings D., Cauchemez S., Fraser C., Riley S., Meeyai A., Iamsirithaworn S., Burke D.S. (2005). Strategies for containing an emerging influenza pandemic in Southeast Asia. Nature.

[38] Thompson W.W., Shay D.K., Weintraub E., Brammer L., Cox N., Anderson L.J., Fukuda K. (2003). Mortality associated with influenza and respiratory syncytial virus in the United States. JAMA.

[39] Wilta F., Chong A.L.C., Selvachandran G., Kotecha K., Ding W. (2022). Generalized Susceptible-Exposed-Infectious-Recovered model and its contributing factors for analysing the death and recovery rates of the COVID-19 pandemic. Appl. Soft Comput..

[40] Cooper I., Mondal A., Antonopoulos C.G. (2020). A SIR model assumption for the spread of COVID-19 in different communities. Chaos Solitons Fractals.

[41] Schultze J.L., Aschenbrenner A.C. (2021). COVID-19 and the human innate immune system. Cell.

[42] Pawelka E., Karolyi M., Mader T., Omid S., Kelani H., Baumgartner S., Ely S., Hoepler W., Jilma B., Koenig F. (2021). COVID-19 is not “just another flu”: A real-life comparison of severe COVID-19 and influenza in hospitalized patients in Vienna, Austria. Infection.

[43] Liu X., Li F., Niu H., Ma L., Chen J., Zhang Y., Peng L., Gan C., Ma X., Zhu B. (2019). IL-2 Restores T-Cell Dysfunction Induced by Persistent Mycobacterium tuberculosis Antigen Stimulation. Front. Immunol..

[44] Saeidi A., Zandi K., Cheok Y.Y., Saeidi H., Wong W.F., Lee C.Y.Q., Cheong H.C., Yong Y.K., Larsson M., Shankar E.M. (2018). T-Cell Exhaustion in Chronic Infections: Reversing the State of Exhaustion and Reinvigorating Optimal Protective Immune Responses. Front. Immunol..

[45] Palmer C.S. (2022). Innate metabolic responses against viral infections. Nat. Metab..

[46] Sumbria D., Berber E., Mathayan M., Rouse B.T. (2021). Virus Infections and Host Metabolism—Can We Manage the Interactions?. Front. Immunol..

[47] Yu L., Chen X., Wang L., Chen S. (2018). Oncogenic virus-induced aerobic glycolysis and tumorigenesis. J. Cancer.

[48] Li H., Zhao A., Li M., Shi L., Han Q., Hou Z. (2022). Targeting T-cell metabolism to boost immune checkpoint inhibitor therapy. Front. Immunol..

[49] Stirling E.R., Bronson S.M., Mackert J.D., Cook K.L., Triozzi P.L., Soto-Pantoja D.R. (2022). Metabolic Implications of Immune Checkpoint Proteins in Cancer. Cells.

[50] Chaudhary O., Trotta D., Wang K., Wang X., Chu X., Bradley C., Okulicz J., Maves R.C., Kronmann K., Schofield C.M. (2022). Patients with HIV-associated cancers have evidence of increased T cell dysfunction and exhaustion prior to cancer diagnosis. J. Immunother. Cancer.

[51] Cai H., Liu G., Zhong J., Zheng K., Xiao H., Li C., Song X., Li Y., Xu C., Wu H. (2020). Immune Checkpoints in Viral Infections. Viruses.

[52] Bayati F., Mohammadi M., Valadi M., Jamshidi S., Foma A.M., Sharif-Paghaleh E. (2021). The Therapeutic Potential of Regulatory T Cells: Challenges and Opportunities. Front. Immunol..

[53] Baron M., Soulié C., Lavolé A., Assoumou L., Abbar B., Fouquet B., Rousseau A., Veyri M., Samri A., Makinson A. (2022). Impact of Anti PD-1 Immunotherapy on HIV Reservoir and Anti-Viral Immune Responses in People Living with HIV and Cancer. Cells.

[54] Macatangay B.J.C., Gandhi R.T., Jones R.B., Mcmahon D.K., Lalama C.M., Bosch R.J., Cyktor J.C., Thomas A.S., Borowski L., Riddler S.A. (2020). T cells with high PD-1 expression are associated with lower HIV-specific immune responses despite long-term antiretroviral therapy. AIDS.

[55] Velu V., Titanji K., Zhu B., Husain S., Pladevega A., Lai L., Vanderford T.H., Chennareddi L., Silvestri G., Freeman G.J. (2009). Enhancing SIV-Specific Immunity In Vivo by PD-1 Blockade. Nature.

[56] Binder B. (2020). Thimme RCD4+ T cell responses in human viral infection: Lessons from hepatitis C. J. Clin. Investig..

[57] Nakamoto N., Kaplan D.E., Coleclough J., Li Y., Valiga M.E., Kaminski M., Shaked A., Olthoff K., Gostick E., Price D.A. (2008). Functional restoration of HCV-specific CD8 T cells by PD-1 blockade is defined by PD-1 expression and compartmentalization. Gastroenterology.

[58] Bengsch B., Johnson A.L., Kurachi M., Odorizzi P.M., Pauken K.E., Attanasio J., Stelekati E., McLane L.M., Paley M.A., Delgoffe G.M. (2016). Bioenergetic insufficiencies due to metabolic alterations regulated by PD-1 are an early driver of CD8+ T cell exhaustion. Immunity.

[59] Puigserver P., Spiegelman B.M. (2003). Peroxisome proliferator-activated receptor-gamma coactivator 1 alpha (PGC-1 alpha): Transcriptional coactivator and metabolic regulator. Endocr. Rev..

[60] Schank M., Zhao J., Moorman J.P., Yao Z.Q. (2021). The Impact of HIV- and ART-Induced Mitochondrial Dysfunction in Cellular Senescence and Aging. Cells.

[61] Sena L.A., Li S., Jairaman A., Prakriya M., Ezponda T., Hildeman D.A., Wang C.R., Schumacker P.T., Licht J.D., Perlman H. (2013). Mitochondria are required for antigen-specific T cell activation through reactive oxygen species signaling. Immunity.

[62] Schurich A., Pallett L.J., Jajbhay D., Wijngaarden J., Otano I., Gill U.S., Hansi N., Kennedy P.T., Nastouli E., Gilson R. (2016). Distinct Metabolic Requirements of Exhausted and Functional Virus-Specific CD8 T Cells in the Same Host. Cell Rep..

[63] Barili V., Boni C., Rossi M., Vecchi A., Zecca A., Penna A., Missale G., Ferrari C., Fisicaro P. (2021). Metabolic regulation of the HBV-specific T cell function. Antivir. Res..

[64] Zeng Z., Wei F., Ren X. (2020). Exhausted T cells and epigenetic status. Cancer Biol. Med..

[65] Xu L., Yao D., Tan J., He Z., Yu Z., Chen J., Luo G., Wang C., Zhou F., Zha X. (2018). Memory T cells skew toward terminal differentiation in the CD8+ T cell population in patients with acute myeloid leukemia. J. Hematol. Oncol..

[66] Pennock N.D., White J.T., Cross E.W., Cheney E.E., Tamburini B.A., Kedl R.M. (2013). T cell responses: Naïve to memory and everything in between. Adv. Physiol. Educ..

[67] Schillebeeckx I., Earls J., Flanagan K.C., Hiken J., Bode A., Armstrong J.R., Messina D.N., Adkins D., Ley J., Alborelli I. (2022). T cell subtype profiling measures exhaustion and predicts anti-PD-1 response. Sci Rep..

[68] Li H., van der Merwe P.A., Sivakumar S. (2022). Biomarkers of response to PD-1 pathway blockade. Br. J. Cancer.

[69] Davis J.E., Sharpe C., Mason K., Tam C.S., Koldej R.M., Ritchie D.S. (2021). Ibrutinib protects T cells in patients with CLL from proliferation-induced senescence. J. Transl. Med..

[70] Porichis F., Hart M.G., Zupkosky J., Barblu L., Kwon D.S., McMullen A., Brennan T., Ahmed R., Freeman G.J., Kavanagh D.G. (2014). Differential impact of PD-1 and/or interleukin-10 blockade on HIV-1-specific CD4 T cell and antigen-presenting cell functions. J. Virol..

[71] Ejrnaes M., Filippi C.M., Martinic M.M., Ling E.M., Togher L.M., Crotty S., von Herrath M.G. (2006). Resolution of a chronic viral infection after interleukin-10 receptor blockade. J. Exp. Med..

[72] Braun M.Y. (2021). The Natural History of T Cell Metabolism. Int. J. Mol. Sci..

[73] Corrado M., Pearce E.L. (2022). Targeting memory T cell metabolism to improve immunity. J Clin Investig..

